# Safe surgical technique: intramedullary nail fixation of tibial shaft fractures

**DOI:** 10.1186/s13037-015-0086-1

**Published:** 2015-12-12

**Authors:** Boris A. Zelle, Guilherme Boni

**Affiliations:** Department of Orthopaedic Surgery, Division of Orthopaedic Traumatology, University of Texas Health Science Center at San Antonio, 7703 Floyd Curl Dr, MC-7774, San Antonio, TX 78229 USA; Department of Orthopaedics and Traumatology, Federal University of São Paulo, Rua Borges Lagoa, 783-50 Andar, São Paulo, 04038032 Brazil

**Keywords:** Tibia, Fracture, Intramedullary, Nail, Knee pain

## Abstract

Statically locked, reamed intramedullary nailing remains the standard treatment for displaced tibial shaft fractures. Establishing an appropriate starting point is a crucial part of the surgical procedure. Recently, suprapatellar nailing in the semi-extended position has been suggested as a safe and effective surgical technique. Numerous reduction techiques are available to achieve an anatomic fracture alignment and the treating surgeon should be familiar with these maneuvers. Open reduction techniques should be considered if anatomic fracture alignment cannot be achieved by closed means. Favorable union rates above 90 % can be achieved by both reamed and unreamed intramedullary nailing. Despite favorable union rates, patients continue to have functional long-term impairments. In particular, anterior knee pain remains a common complaint following intramedullary tibial nailing. Malrotation remains a commonly reported complication after tibial nailing. The effect of postoperative tibial malalignment on the clinical and radiographic outcome requires further investigation.

## Background

Intramedullary nail fixation remains the treatment of choice for unstable and displaced tibial shaft fractures in the adult [1]. The goals of surgical treatment are to achieve osseous union and to restore length, alignment, and rotation of the fractured tibia. Intramedullary nailing carries the advantage of minimal surgical dissection with appropriate preservation of blood supply to the fracture. Moreover, the surgical implant offers appropriate biomechanical fracture stabilization and acts as a load sharing device allowing for early postoperative mobilization. Recent advances in nail design and reduction techniques have expanded the indications for intramedullary nail fixation to include proximal and distal third tibial fractures.

As of today, intramedullary nail fixation represents a well-described and commonly performed surgical procedure for both the community orthopaedic surgeon as well as the subspecialized orthopaedic trauma surgeon. Despite its popularity, intramedullary nail fixation of displaced tibial shaft fractures remains challenging and is associated with multiple potential pitfalls. The surgical technique continues to evolve and numerous recent investigations have contributed significant advances in this area. The goal of this article is to describe the current concepts of intramedullary nail fixation of tibial shaft fractures and to summarize recent developments in this field.

## Evaluation and initial management

In younger patients, tibial shaft fractures are frequently the result of high-energy injuries and patients must be evaluated for associated injuries according to Advanced Trauma Life Support (ATLS) guidelines. The injured lower extremity must be examined in a thorough fashion. Injuries to the surrounding skin and soft tissues, such as fracture blisters, skin abrasions, burns, ecchymosis or skin tenting, must be recorded and documented. Open fractures must be identified and appropriate tetanus update and antibiotics should be initiated immediately upon the initial presentation. A comprehensive neurovascular examination must be performed and documented.

**Fig. 1:**
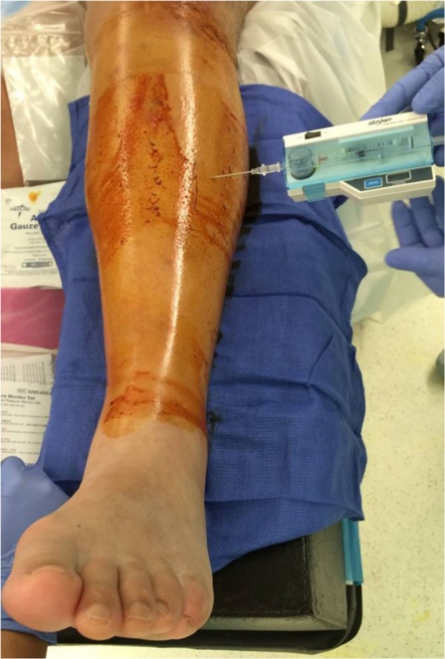
Compartment pressure measurement of the right leg anterior muscle compartment with a pressure needle

The evaluating surgeon should maintain a high suspicion for an associated compartment syndrome and serial clinical examinations are required in these patients. Recent investigations have shown that in diaphyseal tibial fractures the rate of associated compartment syndrome may be as high as 11.5 % [2]. In particular, the younger patient population seems to be at increased risk for the development of a compartment syndrome [2, 3]. The diagnosis of a compartment syndrome should be based on clinical findings including pain, use of narcotics, neurovascular changes, swelling of the muscle compartments, and pain increase with passive toe stretch. Thus, compartment syndrome remains a clinical diagnosis and a thorough documentation of the clinical examination is crucial. Measuring of intracompartmental pressures through a pressure needle (Fig. 1) has been suggested as a useful tool and may play a role, in particular in the obtunded patient when the availability of clinical data points is limited [4–6]. In order to obatin reliable data, the intracompartmental pressures should be measured in all four muscle compartments and in different locations within the respective muscle compartments. A differential pressure (diastolic blood pressure minus compartment pressure) of less than 30 mmHg has been suggested to be indicative of a compartment syndrome [4, 6]. It is important to recognize that diastolic blood pressures typically drop during the surgery and that the preoperative diastolic blood pressure should be considered for calculating the differential pressure [7]. Recent investigations have suggested intracompartmental pressure monitoring as a potentially useful tool for diagnosing acute compartment syndrome with an estimated sensitivity of 94 % and a specificity of 98 % [5]. However, given the potentially devastating consequences of a missed compartment syndrome, we strongly emphasize that the diagnosis of a compartment syndrome should be based on clinical exam findings. In our opinion, the use of intracompartmental pressure measurements should be reserved for special situations, such as the obtunded patient or when clinical data points are equivocal.

The radiographic evaluation of patients with tibial shaft fractures should include standard anteroposterior and lateral radiographs of the injured tibia along with dedicated radiographs of the adjacent knee and ankle joint. Associated tibial plateau fractures should be further evaluated using computer tomography (CT) scans. Similarly, CT scans of the ankle may be required in order to identify and depict fracture lines extending into the tibial plafond as well as associated noncontiguous ankle injuries.

### Pitfalls

**Fig. 2:**
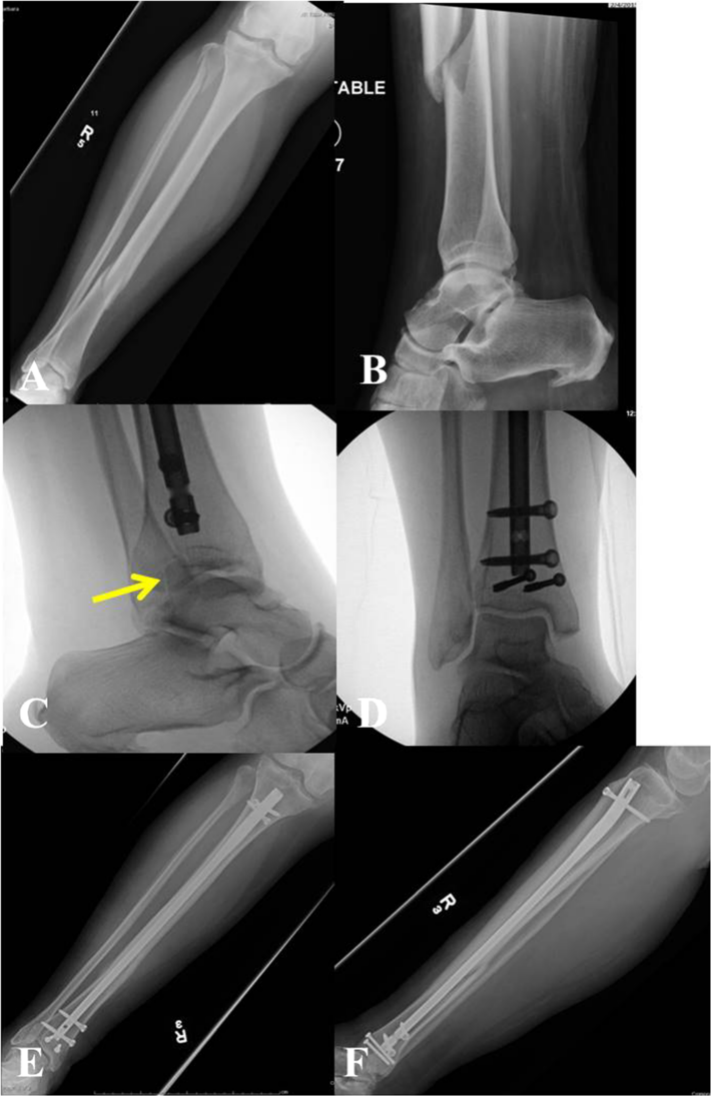
**a**-**f** Right distal third spiral tibia fracture (**a**) with normal appearing preoperative radiographs of the ankle (**b**). Intraoperative fluoroscopic pictures demonstrating a non-contiguous minimally displaced posterior malleolus fracture (**c**) requiring surgical fixation (**d**). Follow-up radiographs (**e**-**f**) show uneventful healing of both tibia and ankle injury

**Fig. 3:**
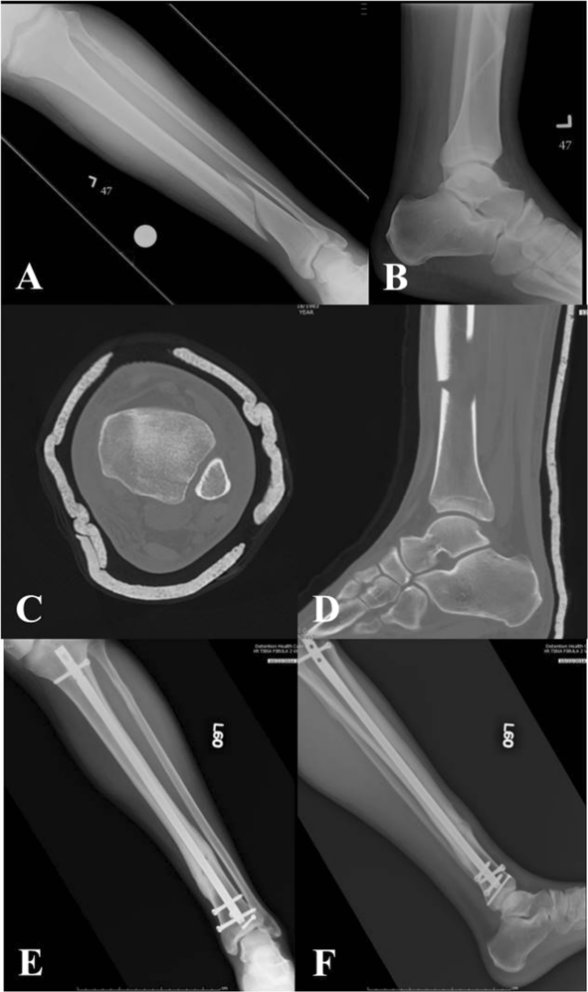
**a**-**f** Left distal third spiral tibia fracture (**a**) with normal appearing radiographs of the ankle (**b**). Preoperative CT scan shows a non-contiguous minimally displaced posterior malleolus fracture (**c**-**d**). Follow-up radiographs (**e**-**f**) show uneventful healing of both tibia and ankle injury

*In particular, distal third tibia fractures have been reported to have a high rate of noncontiguous associated ankle fractures* [8].* Using routine CT scans, Purnell et al.* [8] *reported that 43 % of distal third tibia fractures had associated ankle fractures. The majority of these associated ankle fractures were found to require surgical treatment. The most commonly observed fracture pattern was characterized by a spiral distal third tibial shaft fracture associated with a minimally or non-displaced posterior malleolus fractures (Fig.*2*a**-**f**). Furthermore, the authors of this investigation reported that due to the minmal displacement of the associated ankle fracture, only 45 % of these injuries were identified on the plain radiographs of the ankle by a fellowship-trained orthopaedic traumatologist* [8]*. Therefore, routine CT scans of the ankle should be given a strong consideration in the presence of distal third tibial shaft fractures (Fig.*3*a**-**f**).*

## Surgical considerations

### Tibial nail starting point

Establishing an accurate starting point continues to play a crucial role in any intramedullary nailing procedure. Research studies have provided important information on the anatomic location of the ideal starting point for intramedullary nailing of tibia fractures [9–11]. These investigations demonstrated that the ideal starting point lies at the anterior edge of the tibial plateau and just medial to the lateral tibial spine. Moreover, Tornetta et al. [11] reported on a safe zone with a width of 22.9 mm ± 8.9 mm which allows for a safe nail insertion without the risk of damage to the adjacent articular structures. Traditionally, the starting point for intramedullary nailing of tibial shaft fractures has been established through an infrapatellar approach either by splitting the patellar tendon (transtendinous approach) or alternatively by dissecting just adjacent to the patellar tendon (paratendinous approach). Using this traditional technique, the knee is resting over the radiolucent triangle in a flexed or hyperflexed position. The radiolucent triangle serves as a device to position the leg in a flexed position while the starting point is established. The radiolucent triangle may also assist in applying traction during the reduction maneuver and nail insertion.

**Fig. 4:**
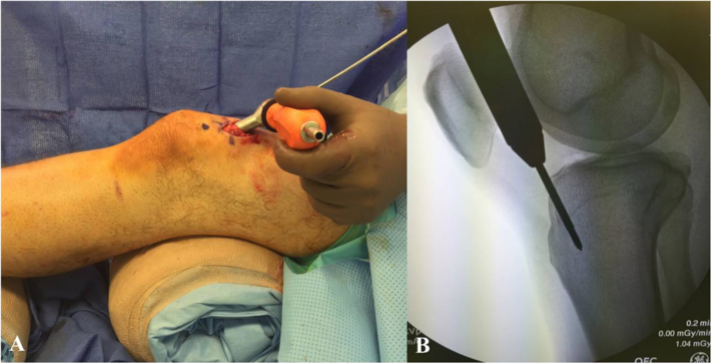
**a**-**b** Intraoperative picture (**a**) demonstrating the suprapatellar starting point through a longitudinal split of the quadriceps tendon and cannula insertion through the patellofemoral joint. Corresponding intraoperative fluoroscopic pictures with lateral view of the starting point (**b**)

Nailing in the semiextended position has recently gained significant attention in the orthopaedic literature [12–15]. Nailing in the semiextended position using a medial parapatellar approach has been suggested by Tornetta and Collins as a method to avoid apex anterior deformities [16]. Recent reports have adopted this concept suggesting tibial nailing in the semiextended position using a suprapatellar portal and nail insertion through the patellofemoral joint [14, 15]. Over the last years, surgical instrumentation has been developed for this technique in order to allow the procedure to be performed in a safe fashion and with minimal damage to the adjacent intraarticular structures. The procedure is performed with the knee flexed approximately 15–20 degrees. An approximately 3 cm longitudinal incision is made about one to two fingerbreadths above the patella. The quadricepts tendon is split in a longitudinal fashion and the patellofemoral joint is entered through further blunt dissection. A cannula system with a blunt trochar is then inserted through the patellofemoral joint in order to establish the starting point at the junction of the anterior cortex of the proximal tibia and the articular surface (Fig. 4a-b). The starting point is established under fluoroscopic guidance using a 3.2-mm guide pin strictly adhering to the fluoroscopic landmarks described above. A multiholed guide pin sleeve is available and may allow for fine adjustments of the starting point. The remaining surgical procedure including reaming of the canal and tibial nail insertion is performed through the cannula system which allows for safe protection of the surrounding soft tissues and articular structures.

**Fig. 5:**
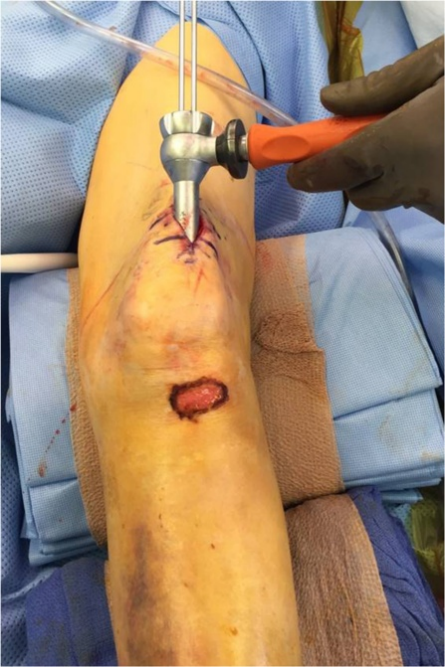
Intraoperative picture demonstrating the soft tissue injury to the infrapatellar area as an indication for suprapatellar nailing in the semiextended position

Suprapatellar nailing in the semiextended position offeres several potential advantages. The semiextended leg position potentially facilitates the fracture reduction in particular in proximal third tibial fractures with the typical apex anterior deformity. In these injury patterns, hyperflexion of the knee over the radiolucent triangle may exaggerate the existing apex anterior deformity. In contrast, the semiextended position may eliminate the extension force of the quadriceps and may greatly facilitate the reduction of the apex anterior angulation. Moreover, the leg resting on the operating room table may facilitate the maneuvering of the leg during the surgical procedure and may facilitate the access of the fluoroscopic image intensifier. Suprapatellar nailing in the semiextended position may also represent a feasible alternative to the traditional infrapatellar approach when soft tissue injuries around the infrapatellar area make the placement of surgical incisions undesireable (Fig. 5).

Recently published studies have suggested suprapatellar tibial nailing technique in the semiextended position as a safe and effective surgical technique. However, there certainly remains the concern of iatrogenic damage to structures of the patellofemoral joint. Using a cadaver model, Gelbke et al. [13] measured the contact pressures in the patellofemoral joint during suprapatellar nailing in the semiextended position versus infrapatellar nailing. These authors reported higher peak pressures with the suprapatellar nailing technique. However, the authors also reported that the observed peak pressures were well below the threshold that has been reported to be detrimental to articular cartilage and they concluded that suprapatellar nailing in the semiextended position represents a safe surgical technique [13]. In a prospective clinical study including 56 patients undergoing suprapatellar tibial nailing in the semiextended position, Sanders et al. [15] did not identify any significant sequelae affecting the patellofemoral cartilage as per Magnetic Resonance Imaging and arthroscopic follow-up evaluations. Interestingly, no patient in this series complained of anterior knee pain at the 12 months follow-up. In a retrospective cohort study, Jones et al. [14] recorded the outcomes of 38 patients undergoing suprapatellar nailing in the semiextended position versus 36 patients undergoing infrapatellar nailing. These authors reported no differences in anterior knee pain and no functional differences between the two patient groups at a minimum of 12 months follow-up. Moreover, these investigators reported significantly better fracture reductions and more precise starting points in the suprapatellar nailing group. These promising data suggest that suprapatellar tibial nailing in the semiextended position represents a safe surgical technique and appropriate clinical and radiographic outcomes can be achieved using this approach. However, future clinical trials are required to further study the advantages and disadvantages of suprapatellar nailing and to evaluate the long-term outcomes associated with this technique.

### Reduction techniques

Placement of the tibial nail alone does not result in adequate fracture reduction and appropriate fracture alignment must be maintained throughout the reaming process and nail placement. While application of longitudinal traction typically results in improved fracture alignment through ligamentotaxis, the simple application of manual traction by itself may not always achieve an anatomic fracture alignment. Various closed, minimal invasive, and open reduction maneuvers have been described and should be in the surgeons armamentarium.

#### Technical trick

**Fig. 6:**
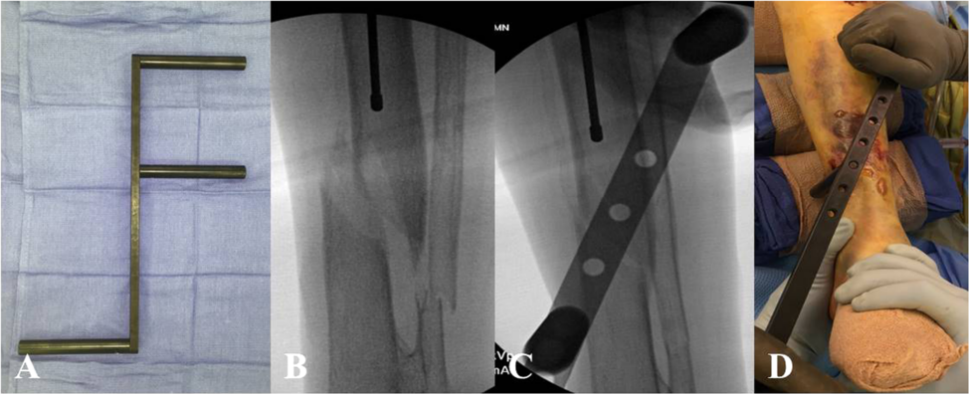
**a**-**d** The F-tool (**a**) allowing for reduction of a medially translated tibia fracture (**b**-**d**)

**Fig. 7:**
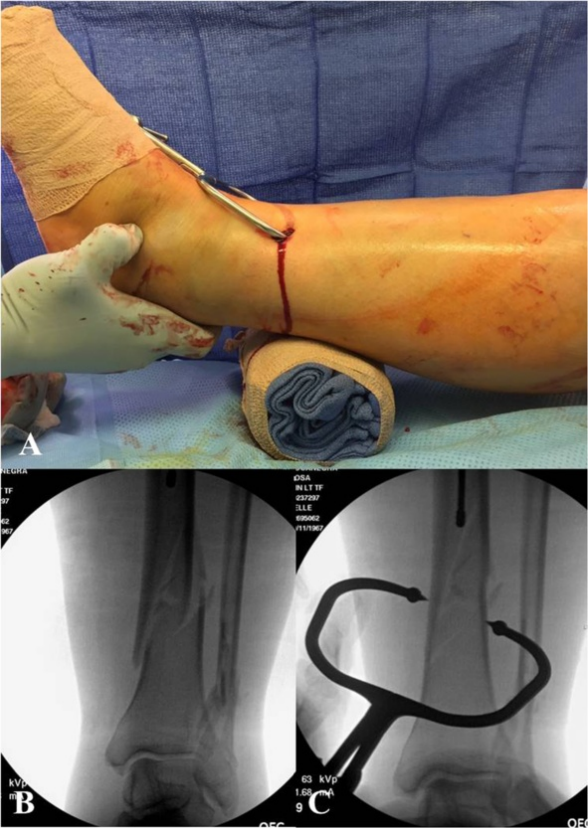
**a**-**c** A percutaneously placed periarticular clamp (**a**) allowing for reduction of a distal third spiral tibia fracture (**b**-**c**)

**Fig. 8:**
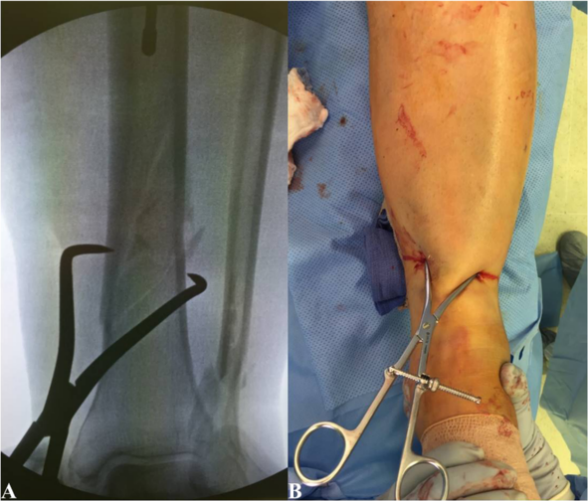
**a**-**b** In same patient, a percutaneously placed pointed reduction clamp (**a**) resulted in significant soft tissue compromise (**b**) which required changing to a different clamp

*Closed reduction maneuvers can be facilitated by widely available reduction tools, such as the F-tool. The F-tool is a an F-shaped radiolucent reduction device that will allow for correction of varus/valgus angulation as well as correction of medial/lateral translation (Fig.**6**a*-*d**). However, due to significant pressure on the tissues prolonged application of this reduction device should be avoided. Certain fractures are also amenable to placement of percutaneously placed reduction clamps. In particular, spiral and oblique fractures lean themselves towards placement of percutaneous clamps. These clamps can be applied in a soft tissue friendly manner through small stab incisions (Fig.*7*a*-*c**). The type of the clamp and the location of the surgical incisions should be strategically chosen in order to minimize any prolonged soft tissue compromise from clamp placement (Fig.*8*a*-*b**).*

The universal distractor can be used as an additional reduction tool [17]. The universal distractor may assist in maintaining length and alignment. Careful attention must be paid to the placement of the Schanz pins. These are placed from the medial side into the proximal and distal fragment away from the planned position of the tibial nail. Moreover, the proximal Schanz pin can be placed in a position that mimics the position of a proximal blocking screw [17]. This may become particularly useful when seeking fracture reduction in proximal tibia fractures with the typical apex anterior deformity. Similar to the universal distractor, two-pin external fixation can be used to obtain and maintain length and alignment during intramedullary nailing of tibial shaft fractures [18]. When using this technique, the pin placement should follow the same principles as with the use of the universal distractor.

In some instances closed and minimal invasivive reduction techniques remain insufficient in obtaining an anatomic fracture alignment. In these cases, open reduction techniques with respectful handling of the surrounding soft tissues should be considered [19, 20]. Open reduction techniques allow for surgical reduction under direct visualization. Potential disadvantages of open reduction techniques include the additional surgical dissection which in may potentially increase the risk of surgical site infection. Moreover, the additional stripping of the blood supply to the fracture site may potentially increase the risk of subsequent fracture nonunion. However, retrospective cohort studies have not shown any increased risk of surgical site infection or fracture nonunion with the use of open reduction techniques [19, 20].

#### Technical trick

**Fig. 9:**
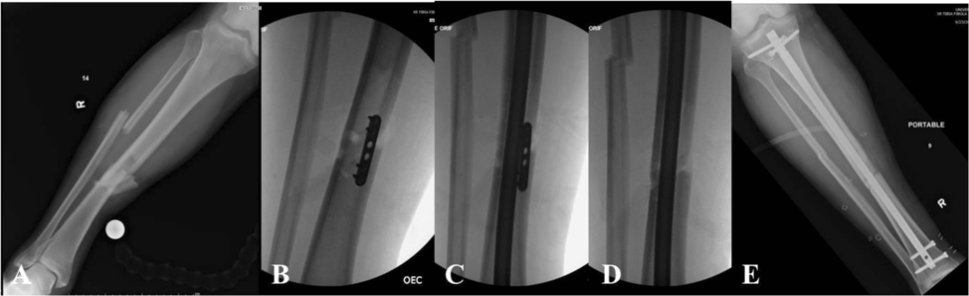
**a**-**e** Open tibia fracture with significant comminution and bone loss (**a**). A unicortical plate was applied through the traumatic wound to achieve fracture reduction (**b**). The plate was maintained throughout the reaming process and nail placement (**c**). Following successful nail stabilization, the plate was removed (**d**-**e**)

*Open reduction maneuvers do not only allow for placement of appropriate surgical reduction clamps, but also provide the opportunity to apply a small- or mini-fragment plate at the fracture site in order to achieve and maintain fracture reduction during the intramedullary nailing procedure* [17, 21]*. The plates are secured to the proximal and distal fracture fragments using unicortical screws. The plate is then maintained throughout the reaming procedure and placement of the intramedullary tibial nail. Following nail placement the plate can be removed or alternatively be left in situ in order to enhance the stability of the fixation construct (Fig.*9*a**-**e**). If the surgeon chooses to leave the plate in situ, the unicortical screws should be exchanged against bicortical screws. Unicortical plating or “reduction plating” has been suggested as a safe and effective technique and should be considered for select cases of tibial shaft that require an open approach to achieve an acceptable fracture reduction* [17, 21]*.*

**Fig. 10:**
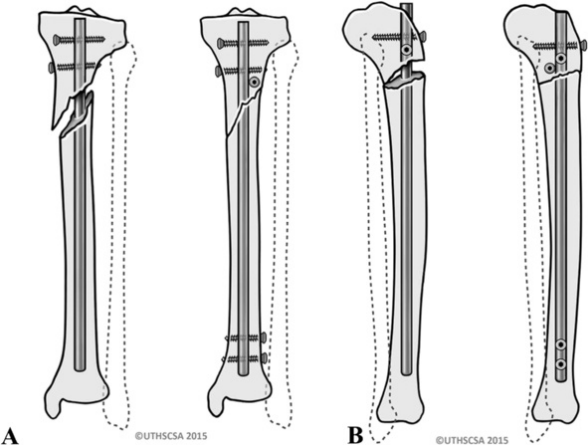
**a**-**b** Blocking screw placed anterior to posterior on the lateral side to prevent valgus deformity (**a**). Blocking screw placed posteriorly from medial to lateral preventing apex anterior deformity (**b**)

Blocking screws (or “poller” screws) have been popularized by Krettek et al. [22]. The purpose of blocking screws is to narrow the canal in the metaphyseal area and to substitute a deficient cortex. Therefore, blocking screws are useful tools in fractures with metaphyseal involvement. The blocking screws are placed prior to the reaming process and nail placement. Blocking screws are typically placed in the short, articular fragment and on the concave side of the deformity. For instance, the typical deformity of a proximal third tibia fracture is characterized by a valgus- and apex anterior deformity. In order to overcome the valgus deformity, a blocking screw can be placed in an anterior to posterior direction into the lateral portion of the proximal fracture fragment (i.e. on the concave side of the deformity). This blocking screw is used to guide the nail medially and thus prevents a valgus angulation. Similarly, the apex anterior deformity can be overcome by a blocking screw that is placed in a medial to lateral direction in the posterior portion of the proximal fragment (i.e. on the concave side of the deformity) (Fig. 10a-b). Krettek et al. [22] reported on 21 tibial fractures treated with intramedullary tibial nailing plus blocking screws. These authors reported favorable clinical and radiological outcomes and no complications related to the placement of blocking screws. Ricci et al. [23] reported on 12 patients underoing tibial nailing in conjunction with blocking screws. All but one patient went on to fracture union. The authors reported only one patient with an angular deformity of more than 5 degrees. This patient was found to have a postoperative valgus angulation of 10 degrees. However, this patient had not undergone blocking screw placement to control for valgus angulation.

### Reaming of the intramedullary canal

**Fig. 11:**
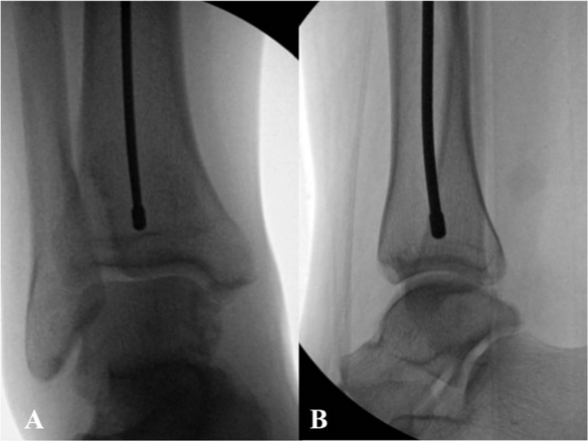
**a**-**b** Anteroposterior (**a**) and lateral (**b**) fluoroscopic pictures demonstrating center/center position of the ball-tipped guidewire

Upon successful completion of the fracture reduction, the intramedullary cavity is prepared for the placement of the tibial nail. A ball-tipped guide wire is typically inserted into the tibial canal and across the fracture site. The reamers as well as the tibial nail are passed over the ball-tipped guide wire. Therefore, it is very important to confirm on fluoroscopic images that the ball-tipped guide wire is positioned appropriately. In particular, it is crucial to confirm that on the level of the ankle joint, the ball-tipped guide wire is well-centered both on the anteroposterior as well as the lateral view (Fig. 11a-b). Following appropriate placement of the ball-tipped guide wire, the reaming process is initiated to prepare the intramedullary cavity for the nail placement.

It appears that at many academic trauma centers reamed tibial nailing is preferred over unreamed tibial nailing [1]. However, the issue of reamed versus unreamed tibial nailing has been discussed controversially. It has been suggested that reamed nailing allows for placement of larger size nails allowing for increased biomechanical stability and potentially improved fracture healing [24]. In contrast, it has been reported that intramedullary reaming results in significant compromise of the endosteal blood supply which may potentially limit the biologic healing response at the fracture site [25]. Moreover, the concern remains that the reaming process may increase the risk of fat embolization and pulmonary failure [26, 27].

Several prospective randomized clinical trials have compared reamed versus unreamed tibial nailing [1, 24, 28–33]. In 2008, the Study to Prospectively Evaluate Reamed Intramedullary Nails in Patients with Tibial Fractures (SPRINT) was published [1]. With a total of 1319 enrolled subjects, this study represents one of the largest prospective randomized clinical trials in the orthopaedic literature overall. These authors reported that among all fractures the risk of a primary event (re-operation and/or autodynamization) was not significantly different between reamed and unreamed tibial nailing. A subgroup analysis showed no differences between the two treatment groups in open tibial fractures. In closed tibial fractures, the risk of a primary event was significantly higher for unreamed tibial nailing. However, this difference was largely driven by the least important outcomes, dynamization and autodynamization. Moreover, the authors reported that the treating surgeons had relatively more experience with reamed tibial nailing. With regards to adverse events, the authors recorded a significantly higher death rate in reamed tibial nailing. The investigators noted that blinded adjudicators classified all deaths as unrelated to the intramedullary nailing procedure [1]. Subsequent meta-analyses as well as a Cochrane review were published with the intent to obtain pooled results from the above mentioned randomized clinical trials [34–37]. The results of these meta-analyses were mostly dominated by the results from the SPRINT study [2] due to its large sample size. Therefore, the results of the above mentioned meta-analyses [34–37] were overall in line with the results from the SPRINT study [1] and mostly confirmed its findings.

We suggest that most surgeons in North America prefer reamed intramedullary tibial nailing over unreamed nailing. However, both reamed and unreamed intramedullary nailing can be suggested as acceptable standard techniques and good outcomes can be achieved with both of these methods.

### Placement of interlocking screws

The purpose of interlocking screws in tibial shaft fractures is to prevent shortening and malrotation. The introduction of interlocking screws has expanded the indication for intramedullary tibial nailing to more proximal and distal third tibial shaft fractures with metaphyseal involvement. In fractures involving the metaphyseal area, interlocking screws become more important in maintaining axial alignment due to the absence of a strong nail/cortex interface. As of today, there are no established clinical guidelines that are providing strong recommendations how many proximal and distal interlocking screws are required for the different fracture types. Most literature in this field is limited to biomechanical investigations and published clinical outcome data is limited.

In a human cadaver model simulating proximal tibia fractures treated with intramedullary nailing, Laflamme et al. [38] reported that the construct stability of two transverse proximal interlocking screws can be significantly increased by the addition of two oblique proximal interlocking screws. In a different human cadaver model simulating intramedullary nailing of extraarticular proximal tibia fractures, Hansen et al. [39] compared the biomechanical stability of two versus three proximal interlocking screws. These authors reported significantly greater stability with three proximal interlocking screws. Using a distal tibia fracture model, Chan et al. [40] compared two versus three distal interlocking screws. These investigators suggested that both fixation constructs provided sufficient stability to allow for postoperative weight-bearing. However, the three-screw fixation construct provided significantly greater stability than the two-screw fixation construct [40]. Moreover, recent studies suggested that angle stable interlocking screws may provide greater stability than conventional interlocking screws, which may allow for potentially achieving the same construct stability with a lower number of interlocking screws [41, 42].

Clinical data providing higher level of evidence with regards to the required number and cofiguration of interlocking screws in tibial nailing remains limited. In a retrospective clinical study evaluating the outcomes in distal tibia fractures undergoing intramedullary nailing, Egol et al. [43] observed that placement of two transverse distal interlocking screws (with or without additional interlocking screws) was associated with less postoperative loss of reduction as compared with other distal interlocking screw constructs. However, in this investigation multiple different screw constructs were chosen and surgical fixation of the associated fibula fracture was at the discretion of the treating surgeon [43]. In a prospective randomized clinical trial in patients with tibial shaft fractures undergoing intramedullary nailing, Kneifel et al. [44] compared one versus two distal interlocking screws. These authors reported a significantly higher rate of screw failure with one distal interlocking screw. With the numbers available no differences with regards to nonunion were found between the two groups [44].

**Fig. 12:**
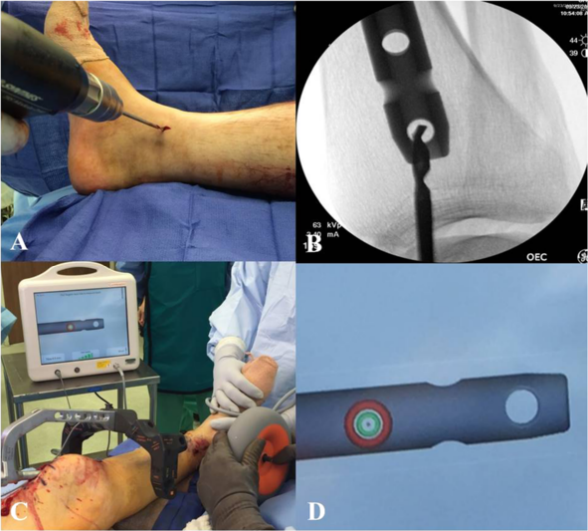
**a**-**d** Placement of distal interlocking screws through fluoroscopic imaging (**a**-**b**) versus electromagnetic guidance system (**c**-**d**)

The placement of proximal interlocking screws is typically performed with the use of an aiming jig that is attached to the nail. The distal interlocking screws are most commonly inserted in a freehand technique under fluoroscopic guidance. Recently, insertion of distal tibial interlocking screws using electromagnetic computer assisted guidance systems has been suggested (Fig. 12a-d) [45–48]. This technique allows for radiation free insertion of distal interlocking screws and has demonstrated to be a feasible and precise method. However, the practical use and cost efficiency of this technique remains to be seen and will require further investigation.

Placement of proximal and distal interlocking screws represents a safe surgical step. However, appropriate awareness of the surrounding anatomic structures is required and the insertion of interlocking screws must be performed in a precise and soft tissue friendly manner.

#### Pitfall

*Anatomic studies have demonstrated that in particular with placement of proximal medial-to-lateral oblique interlocking screws there remains a risk of common peroneal nerve palsy* [49]*. In order to minimize this risk, surgeons should consider drilling for the screw under fluoroscopic guidance with the fluoroscopic image intensifier angled perpendicular to the plane of the drill bit as opposed to standard anteroposterior and lateral views. Surgeons should be aware of the relatively thin cortical bone within the proximal tibia and should be conscientious about the fact that penetration of the far tibial cortex by the drill bit may be difficult to appreciate by tactile feedback. Moreover, the close proximity of the fibular head may obscure the tactile impression and leave the surgeon with the impression of being ‘in the bone’ when in fact the fibular head is penetrated. The screw length should not only be determined by the scaled drill, but also by appropriate depth gauge measurements. Any drilling or screw length measurements past 60 mm should raise the suspicion for posterolateral prominence which may put the common peroneal nerve at risk for injury* [49]*.*

#### Pitfall

*With regards to placement of distal anterior-to-posterior interlocking screws, Bono et al.* [50] *emphasized the close proximity of the anterior neurovascular bundle, the anterior tibial tendon, and the extensor hallucis longus. These authors recommended placement of surgical incision and careful soft tissue dissection in order to protect the surrounding neurovascular structures during interlocking screw placement* [50]*.*

**Fig. 13:**
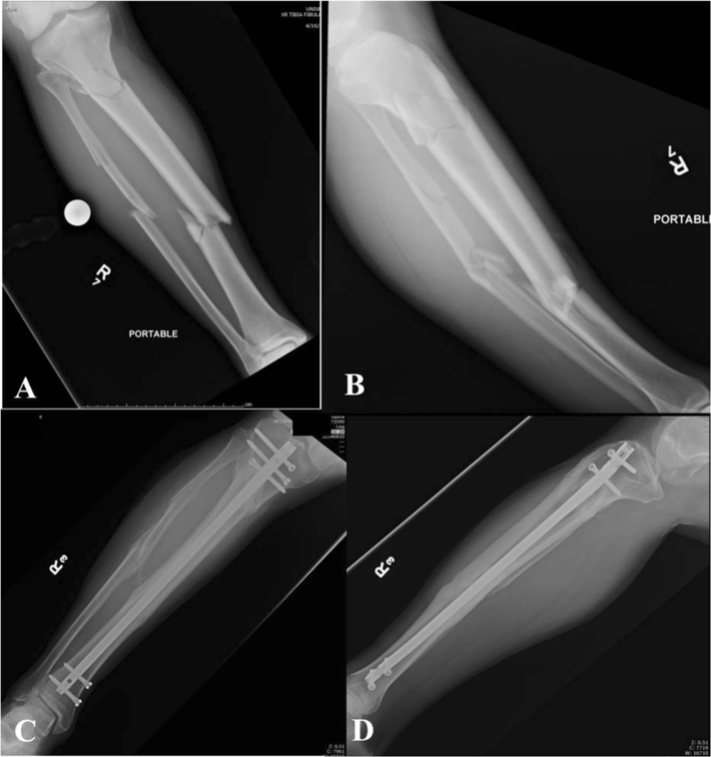
**a**-**d** Segmental tibia fracture (**a**-**b**) treated with intramedullary nailing with two distal and three proximal interlocking screws. Follow-up radiographs (**c**-**d**) demonstrate uneventful healing

We therefore suggest placement of interlocking screws as an important part of the intramedullary nailing procedure. While percutaneous screw placement is typically safe, surgeons need to be aware of the surrounding soft tissue structures at risk. For most tibial shaft fractures two proximal and two distal interlocking screws provide sufficient stability. Proximal and distal third tibial fractures may benefit from placement of additional interlocking screws in different planes in order to increase the stability of the construct (Fig. 13a-d).

### Fixation of associated fibula fractures

Contemporary nail designs with distal interlocking screw options have expanded the indication of intramedullary tibial nailing to include proximal and distal fractures involving the metaphyseal area. With regards to distal metaphyseal fractures the question remains if an associated distal fibula fracture should be treated with or without surgical fixation. Currently, there is no consensus in the literature with regards to this issue.

In 2006, Egol et al. [43] reported on 72 distal tibia fractures undergoing intramedullary tibial nail fixation that were associated with a fibula fracture. In 25 cases, surgical fixation of the fibula was performed. In 47 cases, the associated fibula fracture was treated without surgical fixation. The decision for fibula stabilization was at the discretion of the treating surgeon. Various distal interlocking screw constructs were used in this study (2 screws from medial to lateral versus 2 screws placed perpendicular to each other versus total of 3 distal interlocking screws versus only one distal interlocking screw). The authors reported that loss of reduction was significantly lower in patients receiving fibula stabilization in conjunction with intramedullary tibial nail fixation. In patients undergeoing intramedullary nail fixation without fibula stabilization, a total of 13 % showed postoperative loss of reduction versus 4 % when tibial nailing was performed without fibula stabilization. The authors further reported that two medial to lateral distal interlocking screws seemed to prevent postoperative loss of reduction, but this finding was not statistically significant. It must be pointed out that in the fibula stabilization group, the authors recorded a significantly higher percentage of patients with the potentially more favorable distal interlocking screw construct (2 medial to lateral screws with or without anteroposterior screw) than in the no fibula stabilization group (86 % versus 45 % of fractures). In addition, it was recorded that the more distal fractures were more likely to receive fibula stabilization. Thus, the results of this investigation did not seem controlled for fracture location, configuration of distal interlocking screws, and number of distal interlocking screws [43].

In a prospective randomized clinical trial, Prasad et al. [51] compared intramedullary tibial nail fixation with fibula fixation versus intramemedullary tibial nail fixation without fibula fixation in 60 distal third tibia-fibula fractures. The authors reported improved rotational and varus/valgus alignment in patients undergoing fibula fixation in conjunction with tibial nailing. However, the authors also reported a wound complication rate of 10 % in the fibula fixation group [51].

We conclude that in distal third tibial shaft fractures undergoing intramedullary nail fixation, adjunct fibula fixation may allow for achieving and maintaining fracture reduction of the tibia. However, there remains the concern of wound complications from the additional incision in the area of traumatized tissue. We therefore suggest using adjunct fibula fixation cautiously. Contemporary tibial nail designs typically provide different options for placement of stable distal interlocking screw constructs minimizing the risk for postoperative loss of reduction. Additional plate fixation of the fibula should be reserved for associated unstable injuries to the ankle joint or when it is felt that anatomic tibial alignment cannot be achieved without direct reduction of the associated fibula fracture.

## Outcomes

Good outcomes and reproducible results can be achieved with intramedullary nail fixation of tibial shaft fractures. The reported union rates of intramedullary tibial nailing vary among different studies. With contemporary implants and appropriate surgical techniques, union rates above 90 % can be expected [34–37]. Tibial shaft fractures that fail to heal following intramedullary nail fixation typically respond well to exchange reamed nailing procedures [52].

Despite favorable union rates that can be achieved with intramedullary nail fixation of tibial shaft fractures, patients continue to have functional long-term sequelae following this procedure. Outcome evaluations at one year after surgery demonstrated that as many as 44 % of patients continued to have functional limitations with regards to their injured lower extremity [53]. Moreover, it has been reported that at one year after surgery as many as 47 % of patients continue to report work-related disability [54]. Other follow-up studies recorded that at approximately two years after intramedullary nail fixation, almost 20 % of patients had not yet returned to their previous occupation and almost 30 % had not yet returned to their previous level of recreation [31]. In a long-term outcome study including 56 patients after tibial nailing with a median follow-up of 14 years, Lefaivre et al. [55] reported that the SF-36 and the Short Musculoskeletal Functional Assessment (SMFA) scores were not statistically different from reference population norms. However, 73.2 % of patients self-reported at least moderate knee pain and 33.9 % of patients self-reported complaints of swelling. The physical examination showed decreased range of motion of the ankle joint in 42.4 % of examined patients while 93.9 % of patients demonstrated full range of motion of the knee joint. Atrophies of the calf and/or the quadriceps muscles were observed in 27.3 % of patients. Radiographic evidence of osteoarthritis of the knee and/or ankle joint was found in 35.4 % of patients despite the absence of radiographic tibial malalignment [55].

These data indicate that patients undergoing intramedullary tibial nailing continue to have remarkable functional limitations in the long-term. Surgeons should be aware of these issues and counsel patients accordingly.

### Anterior knee pain

Anterior knee pain is a commonly reported complication after intramedullary nailing of tibial shaft fractures [55–62]. A comprehensive review with pooled data from publications including the years 1990 until 2005 suggested that postoperative knee pain may occur in approximately 47 % of patients following intramedullary nailing [60]. The exact etiology of anterior knee pain following tibial nailing is not fully understood. Potentially contributing factors may include traumatic and iatrogenic damage to intraarticular structures, injuries to the infrapatellar branch of the saphenous nerve, thigh muscle weakness secondary to pain-related neuromuscular reflex inhibition, fat pad fibrosis leading to impingement, reactive patellar tendonitis, bending strain exerted by the nail on the proximal part of the tibial bone, and proximal protrusion of the nail [10, 57, 58, 60, 63, 64]. As of today, it must be assumed that the reason for postoperative knee pain is multifactorial and that the different above-named factors may be contributing to this problem at varying degrees.

In an attempt to address the etiology of anterior knee pain after intramedullary nailing, transtendinous approaches have been compared to paratendinous approaches. Previous studies suggested that transtendinous may be associated with a higher incidence of postoperative knee pain [61]. However, prospective randomized clinical data has not shown any significant difference between the transtendinous and paratendinous approach [65–67]. In a prospective randomized clinical trial including fifty patients undergoing intramedullary tibial nailing, Toivanen et al. [65] did not find any significant differences in the functional outcomes of the transtendinous versus paratendinous approach at an average follow-up of 3.2 years. In a subsequent follow-up study using the same patient population, these authors reported on the long-term results with an eight year follow-up [66]. At eight year follow-up, there were no significant differences between the two approaches. Of note, these investigators also observed a significant decrease in anterior knee pain over time. While 69 % of patients complained of anterior knee pain at 3.2 years after surgery, a total of 29 % complained of knee pain at the eight year follow-up [66].

The effect of elective hardware removal in order to address anterior knee pain following intramedullary tibial nailing remains uncertain. Court-Brown et al. [56] reported marked or complete relief of anterior knee pain in 60 out of 62 patients who underwent elective tibial nail removal due to persistent anterior knee pain following intramedullary tibial nailing. In contrast, Keating et al. [61] reported on 49 patients undergoing tibial nail removal due to persistent anterior knee pain. These authors reported complete relief in approximately 45 %, partial relief in approximately 35 %, and no improvement in approximately 20 % of patients. Therefore, the indication for tibial nail removal in the treatment of postoperative anterior knee pain remains poorly defined. We suggest considering a tibial nail removal only in patients with persistent anterior knee pain if a mechanical etiology, such as nail protrusion or prominent interlocking screws, can be identied. However, in symptomatic patients with appropriately placed hardware, the benefit of a tibial nail removal remains questionable.

With regards to postoperative anterior knee pain, intriguing results have been reported in preliminary clinical investigations of suprapatellar tibial nailing in the semiextended position. Jones et al. [14] reported no statistical differences with regards to anterior knee pain between patients undergoing suprapatellar versus infrapatellar nailing. However, the authors reported that there was trend toward greater symptomatic knee pain in the infrapatellar group. Furthermore, Sanders et al. [15] reported on 56 consecutive patients undergoing suprapatellar nailing in the semiextended position. These authors did not identify any patients with postoperative anterior knee pain at 12 months follow-up except one patient who presented with peri-incisional pain around the knee [15]. While these preliminary data seem encouraging, there remains the theoretical concern of iatrogenic cartilage damage to the patellofemoral joint associated with this procedure [12, 13]. Therefore, larger clinical investigations with long-term follow-up periods are necessary in order to substantiate the impact of suprapatellar nailing on postoperative anterior knee pain.

### Effect of postoperative malalignment

Posttraumatic osteoarthritis remains an important concern following treatment of tibial shaft fractures with intramedullary nailing. Biomechanical studies have demonstrated that tibial malalignment may result in significant changes of contact pressures in the adjacent ankle and knee joint [68]. However, the clinical effects of tibial malalignment on clinical and functional outcomes continue to be controversial.

Clinical investigations evaluating the long-term clinical and radiographic outcomes after tibial shaft fractures have provided conflicting data with regards to the sequelae of tibial malalignment. In a long-term outcome study with an average of 29 years of follow-up, Merchant and Dietz [69] did not find any significant correlation between tibial malalignment and malfunction of the adjacent ankle or knee joint [69]. In contrast, other investigators reported significant correlations between tibial malalignment and ankle and/or knee malfunction [70, 71]. Similarly, the relationship between tibial malalignment and radiographic signs of posttraumatic arthritis of the adjacent joints remains controversial. While some authors [72] reported a significant correlation between tibial malalignment and radiographic signs of posttraumatic arthritis other investigations did not show any significant correlations between these two variables [69, 70]. Of note, most of these long-term data were derived from patient populations in which the majority of subjects were treated nonoperatively with casting and/or bracing [69–72] and these data may not be extrapolated to patients undergoing intramedullary tibial nailing.

Reports on postoperative malalignment following intramedullary tibial nailing remain limited and the reported case numbers are low [73, 74]. It must be assumed that with contemporary implants and appropriate surgical techniques the rates of malangulation are rather low as compared to nonoperative treatment. However, postoperative malrotation remains a commonly reported concern specific to intramedullary tibial nailing. Unfortunately, the intraoperative assessment of tibial rotation remains challenging. As of today, there is no single one clinical or fluoroscopic method that has been established as the gold standard for judging tibial rotation intraoperatively. Results from sophisticated CT assessments recorded that malrotation following intramedullary tibial nailing may be as 19 % to 41 % [75–78]. In particular, external rotation deformities seem to be more common than internal rotation deformities. In a series of 70 patients with an average follow-up of 58 months, Theriault et al. [78] reported that tibial malrotation did not show any significant correlation with the functional outcomes. The authors also reported that clinical examinations to assess for postoperative malrotation were inaccurate and showed low correlations with CT assessments.

In our opinion, malreduction remains a concern for the long-term outcome of tibial shaft fractures undergoing tibial nailing. Despite the conflicting data with regards to the relationship between malalignment and clinical and radiographic outcomes, we suggest that surgeons should strive to achieve an anatomic fracture alignment in an attempt to control for this variable and to achieve the best possible outcome.

## Conclusions

Statically locked, reamed intramedullary nailing remains the standard treatment for displaced tibial shaft fractures. A correct starting point remains a crucial part of the surgical procedure. Suprapatellar nailing in the semi-extended position has been suggested as a safe and effective procedure and future studies are warranted to further evaluate the risks and benefits of this surgical procedure. The treating surgeon should be familiar with contemporary reduction techniques. Open reduction techniques should be considered if anatomic fracture alignment cannot be achieved by closed means. Favorable union rates above 90 % can be achieved by both reamed and unreamed intramedullary nailing. Despite favorable union rates, patients continue to have functional long-term limitations. In particular, anterior knee pain remains a common complaint following intramedullary tibial nailing. In addition, malrotation remains a commonly reported concern after tibial nailing. As of today, no significant correlation between malrotation and functional outcome has been established in the literature.
